# Distributed Model-Free Bipartite Consensus Tracking for Unknown Heterogeneous Multi-Agent Systems with Switching Topology

**DOI:** 10.3390/s20154164

**Published:** 2020-07-27

**Authors:** Huarong Zhao, Li Peng, Hongnian Yu

**Affiliations:** 1 Research Center of Engineering Applications for IOT, Jiangnan University, Wuxi 214122, China; zhaohuarong@stu.jiangnan.edu.cn; 2 Jiangsu Province Internet of Things Application Technology Key Construction Laboratory, Wuxi Taihu College, Wuxi 214145, China; 3 School of Engineering and the Built Environment, Edinburgh Napier University, Edinburgh EH10 5DT, UK; h.yu@napier.ac.uk

**Keywords:** data driven, multi-agent system, bipartite consensus, switching topologies

## Abstract

This paper proposes a distributed model-free adaptive bipartite consensus tracking (DMFABCT) scheme. The proposed scheme is independent of a precise mathematical model, but can achieve both bipartite time-invariant and time-varying trajectory tracking for unknown dynamic discrete-time heterogeneous multi-agent systems (MASs) with switching topology and coopetition networks. The main innovation of this algorithm is to estimate an equivalent dynamic linearization data model by the pseudo partial derivative (PPD) approach, where only the input–output (I/O) data of each agent is required, and the cooperative interactions among agents are investigated. The rigorous proof of the convergent property is given for DMFABCT, which reveals that the trajectories error can be reduced. Finally, three simulations results show that the novel DMFABCT scheme is effective and robust for unknown heterogeneous discrete-time MASs with switching topologies to complete bipartite consensus tracking tasks.

## 1. Introduction

Multi-agent systems (MASs) and machine learning, two exciting trends in the robotics field, have recently attracted more and more researchers’ attention due to the new epoch of artificial intelligence (AI) [1,2]. How to introduce intelligent algorithms into traditional control theories is one of the hottest and significant research topics. Specifically, utilizing intelligent algorithms to improve the robustness of MASs and reducing the calculation burden of designing controllers [3,4,5] to achieve consensus tracking are two of the challenges we need to address.

In the past half-century, most of the excellent control schemes have been developed based on explicit or implicit mathematical models. Examples are sliding model control, intermittent control, impulse control, and fuzzy control, to name but a few. In addition, most of these control theories were successfully applied to consensus tracking tasks of MASs. In [5], Barbot et al. first introduced the concept of a second-order sliding mode. Many novelty approaches have been developed since then. For instance, a novel sliding-mode-based discrete differentiator was proposed that can estimate the accurate derivatives input of the controlled plant [6], and the output constraint problems are considered in the second-order sliding mode controller designer in [7]. In [8], Xu et al. researched the second-order consensus problems of MASs, where local intermittent information among the agents is utilized to design a distributed adaptive completely intermittent controller to achieve second-order consensus. The impulse control approaches can be seen in [9,10], where the fixed-time quantity consensus, delayed and stochastic perturbation, and second-order consensus are considered to design appropriate properties for MASs. In terms of fuzzy control, the author in [11] designed a mixed controller, which consists of a fuzzy controller and a fuzzy observer, to solve the partly unmeasurable states of controlled systems. It is noteworthy that most traditional control algorithms [5,6,7,8,9,10,11] must consider the dynamics of a controlled system, which is called model-based control (MBC).

However, an accurate model of the plant is hard to obtain, so most MBC approaches are established on the approximate dynamics of systems, which usually are not robust in a partial application. Fortunately, in the past few years, with the development of machine learning, another branch of control theory has been developed that is inspired by machine learning and tries to introduce the leaning approach into traditional theories to avoid the difficulties in acquiring or estimating the dynamics of physical systems. To complete similar control tasks as those solved by MBC schemes, the new control theory works by merely using the interactive information between itself and its external environment, improving the control performance by self-leaning; this is called model-free control (MFC) or data-driven control [2,4].

Recently, several papers [12,13,14,15,16,17,18,19,20,21,22,23,24] have reported on model-free adaptive control (MFAC), interactive learning control (ILC), repetitive learning control (RLC), reinforcement learning (RL), and so on. The consensus tracking problems of MASs were researched in [12] by the MFAC approach, where both the time invariable and varying desired trajectories tracking are archived. Moreover, the further theoretical analysis of MFAC was rigorously presented in [4], which introduces that the MFAC method only needs input/output (I/O) measurement data of a controlled plant, without the need of any explicit mathematical model, Lyapunov stability theory, or key technical lemma to design controllers for various control tasks. ILC is an effective approach for repetitive operating systems, which was developed by many researchers such as in [2,13,14]. In [2], Hui et al. extended the dimension of ILC, which has a time dimension, iteration dimension, and space dimension, to achieve a faster and more precise tracking performance for the MASs’ formation task. In [13], Li et al. studied how to combine the ILC with model predictive control to achieve better performance. The RLC model is utilized to track periodic exogenous signals in continuous processes, which can be seen in [14], where a novel distributed adaptive protocol is investigated for uncertain nonlinear leader–follower MASs to achieve global asymptotic consensus. In [15], Odekunle et al. presented a novel approach to solve the non-zero-sum game output regulation problem for MASs by using RL. In our investigations, we found that another category of MFC methods is based on neural networks (NNs), which have unparalleled approximation abilities for nonlinear dynamics. In [16,17,18], the authors designed actor–critic-based neural networks to approximate the value function and control policy for each agent, respectively, to optimize consensus control performance. It should be pointed out that NNs-based methods need training processes and external testing signals for controller design, which are not convenient. Meanwhile, there are some interesting adaptive schemes in [19,20,21,22,23].

In the aforementioned related studies [5,6,7,8,9,10,11], consensus problems of MASs are based on MBC approaches, while the authors of [12,13,14,15,16,17,18,19,20,21,22,23] employed and developed MFC methods to address consensus or consensus tracking problems for MASs; however, it is still an open and challenging problem for unknown dynamics MASs to achieve consensus tracking. Furthermore, it is obvious from a review of the above literature that MASs consensus control and tracking only consider the cooperation interactions among agents. In fact, we usually find that the two relationships are inseparable from one another in natural or engineering scenarios, for instance, activators and inhibitors in biological systems, teams opposed in a sports match, or duopolistic regimes arising when agents compete for limited resources in economical systems [24]. Hence, to improve the adaptive and autonomous abilities of MASs, the competition relationship needs to be considered, which is becoming a hot research topic. Altafini [25] first explored consensus for MASs with antagonistic interactions, and this specific consensus is called bipartite consensus (BC), which means that agents are assigned to two alliances, where each alliance has a unique sign, but each agent ultimately achieves the same position, velocity, and/or angle. After that, BC sparked the interest of many researchers and has been discussed for MASs with linear, nonlinear, and even heterogeneous dynamics. Moreover, the BC for MASs with Lipschitz-type, second-order, or high-order dynamics is investigated in [24,26,27]. Inspired by the above contributions, several theories have been extended. In [28], a distributed extended state observer is employed to guarantee leader–follower BC for MASs with mismatched unknown disturbance. It is observable that formulating a BC controller is more challenging for high-order MASs than for low-order ones. The BC problem for high-order MASs with input saturation is researched by combining distributed event-triggered control and a low-gain feedback technique in [29]. The finite-time and fixed-time BC for MASs are explored in [30,31], respectively. A novel RL based protocol is presented in [32], which is the first use of RL for unknown discrete-time leader–follower MASs, where the author utilizes data-driven actor–critic-based NNs to address the BC problem for unknown MASs, but it increases computations. Moreover, a training process is necessary.

Although much effort has been made toward solving the BC problem [33,34,35,36], to the best of our knowledge, pseudo partial derivative (PPD) approaches have not been taken into account in the existent results. From the above observations and analysis, this paper employs a PPD method to estimate an equivalent dynamic linearization data model of an easy agent, where merely the measurement I/O data of neighborhood agents is necessary. Then, a distributed model-free adaptive bipartite consensus tracking (DMFABCT) scheme is designed for unknown detected-time heterogeneous nonaffine nonlinear MASs with switching topologies to realize time-invariant and time-varying reference trajectory bipartite consensus tracking tasks by using the neighbor-based tracking error. It is worth pointing out that although a few agents could receive the desired trajectory, the rigorous theoretical proof confirms that our proposed algorithm can guarantee convergence of all agents. In the investigation of the existing consensus approaches of MASs, the main contributions of this work might be summarized as follows:

(1) A DMFABCT framework is established for unknown heterogeneous nonaffine nonlinear detected-time MASs with switching topologies and a coopetition network. It is a data-driven distributed intelligent algorithm, which has good performance to address the BC problem under both time-invariant and time-varying reference trajectories. Although Bu et al. [37] proposed a novel data-driven framework for MASs, it only discussed the cooperative interactions.
(2) The proposed DMFABCT scheme is designed by neighbor-based online measurement I/O data that can bypass the confusion of existing consensus algorithms as seen in [5,6,7,8,9,10,11,24,25,26,27,28,29,30,31,32,33,34,35] to obtain an accurate mathematical model so that the designed scheme is more robust and reduces energy costs from the massive computation.
(3) Both collaborative and antagonistic interactions among agents are considered in the proposed protocol. Compared with the protocols in [1,2,3,4,5,6,7,8,9,10,11,12,13,14,15,16,17,18,19,20,21,22,23], the proposed protocol is more reasonable. Moreover, the difference of DMFABCT from the novel algorithm proposed in [32] is that DMFABCT copes with the BC problem with PPD, where the training processes and external testing signals are not necessary.

The remainder of this paper is structured as follows. Several essential preliminaries are presented in Section 2. The introduction of the DMFABCT algorithm and the tracking performance of fixed and time-varying reference trajectory analysis are presented in Section 3. Three numerical simulation experiments are provided in Section 4. Finally, conclusions and future work are provided in Section 5.

## 2. Preliminaries and Problem Formulation

### 2.1. Graph Theory and Some Notations

Let R denote the set of real numbers. The Euclidean norm of $Χ\in R^{nxn}$ is expressed by ‖Χ‖. The identity matrix and diagonal matrix are expressed by I and diag(•), respectively, where the dimension is dependent on the context. In this paper, the algebraic graph theory is employed to analyze the interaction topologies of MASs. It should to be pointed out that the graphs are directed and the weighted directed graph is expressed by G=(V,E,A), where V={1,2,⋅⋅⋅,N}, $E\subseteq \left\{\left(V_{i}{,V}_{j}\right){|V}_{i}{,V}_{j}\in V\right\}\subseteq V\times V$, and A are the set of vertices, the set of edges, and the adjacency matrix, respectively. Then, i as the parent and j is the child, if the i can transmit the information to j directly, which is expressed as (i,j)∈E. If i is not the father of j, $a_{i,j}=0$, otherwise $a_{i,j}\neq 0$. In the graph of MASs, the i has many children so utilizes the $N_{i}=\left(j|j\neq i,\;\left(V_{j},V_{i}\right)\in E\right)$ to describe the relationships among each agent, which is named as the neighborhood of the agent i in other literature. In this paper, the cooperative and competitive relationships are considered between each agent so that the elements of ${A=(a}_{i,j})\in R^{N\times N}$ have three different values, −1, 0, and 1. If the node i and j belong to a same group, agent i could get the information from agent j, $a_{i,j}=1$, otherwise $a_{i,j}\neq 1$. When $a_{i,j}=-1$, the agents i and j must be in opposite groups, which is called a competitive relationship between the agents i and j. Alternatively, there is another definition, which is cooperation. Moreover, we usually use cooperation to represent the two different situations among the MASs network. The Laplacian matrix of G can be calculated by L=D−A, where $D=diag\left(d_{1}^{in},d_{1}^{in},\cdot \cdot \cdot ,d_{N}^{in}\right)$ and $d_{i}^{in}=\sum_{j=1}^{N}a_{i,j}$ are called in-degree of vertex i. The coopetition network G is called structurally balanced if the whole nodes in V can be divided into two disjointed subsets, that is, $V_{1}$, $V_{2}$. They satisfy the following three conditions:

(1). ${V=V}_{1}\cup V_{2}$ and $V_{1}\cap V_{2}=\emptyset$.
(2). if $\forall i,j\in V_{z}\left(z\in \left\{1,2\right\}\right)$, $a_{ij}\geq 0$.
(3). if $\forall i\in V_{z},j\in V_{q},z\neq q\left(z,q\in \left\{1,2\right\}\right)$, $a_{ij}\leq 0$.

Furthermore, if this MASs graph G contains a spanning tree, the information can transmit from a root node to any other node, and so this graph is considered to be a strongly connected graph.

In order to investigate time-varying switching topologies, let $\bar{G}\left(k\right)$ denote a time-varying switching graph with a virtual leader, which is dependent on k, and $A_{F}\left(k\right)=\left[a_{i}{}_{j}\left(k\right)\right]\in \;R^{N\times N}$, $d_{i}\left(k\right)=\sum_{j\in N(i)}\left|a_{\mathrm{ij}}\left(k\right)\right|$, $L\left(k\right)=-A_{F}\left(k\right)+D\left(k\right)\in R^{N\times N}$ are the corresponding adjacency matrix, degree matrix, and Laplacian matrix, respectively. $N_{p}\left(i\right)$ denotes the neighborhood of the ith agent and $B\left(k\right)=diag\left\{b_{1}\left(k\right),\cdot \cdot \cdot ,b_{N}\left(k\right)\right\}\in R^{N\times N}$ is employed to depict the relationship between the virtual leader 0 and each follower. If the agent i can directly get the desired trajectory from virtual leader 0, $i.e.,\left\{0,i\right\}\in \bar{E}$, $b_{i}\left(k\right)=1$. Otherwise, $b_{i}\left(k\right)=0$. To describe the time-varying topology, let ${\bar{G}}_{l}=\left\{{\bar{G}}_{1},{\bar{G}}_{2},\cdot \cdot \cdot ,{\bar{G}}_{\kappa }\right\}$ denote the set of all directed graphs for the agents, where $\kappa \in Z^{+}$ denotes the total number of possible interaction graphs.

### 2.2. Problem Formulation

In existing studies, the consensus problem, especially the bipartite consensus problem, is often considered for a group of agents with identical dynamics. However, heterogeneity is the intrinsic property for multi-agent systems. Therefore, the problem of bipartite consensus for heterogeneous agents presents many challenges. It is noteworthy that the following assumptions are fundamental conditions of nonlinear dynamics for our analysis.

**Definition** **1.** *Consider a discrete-time heterogeneous SISO (simple-input-simple-output) MAS with N agents and the nonlinear dynamics of agent i satisfies the following equivalent:*

(1) $$y_{i}(k+1)\;=\;f_{i}(y_{i}(k),u_{i}(k))$$

*where $y_{i}\left(k\right)\in R$ is the output, i=1,2,…,N, $f_{i}\left(\cdot \right)$ is an unknown nonlinear function, and $u_{i}\left(k\right)\in R$ is the controlling input, respectively. $y_{0}\left(k\right)$ denotes the trajectory of a virtual leader, which is represented by using vertex 0 in the graph. Furthermore, only a subset of agents can receive information from the virtual leader directly. Hence, the directed graph $\bar{G}$ of MASs is combined with N+1 agents and the corresponding edge set and weighted adjacency matrix are expressed by $\bar{E}$ and $\bar{A}$, respectively.*

**Assumption** **1.** *$u_{i}\left(k\right)$ is a continuous function in order to obtain the partial derivative of nonlinear function $f_{i}\left(\cdot \right)$.*

**Assumption** **2.** *Those conditions where $u_{i}\left(k-1\right)$, $\Delta u_{i}\left(k\right)\neq 0$, $\left|\Delta y_{i}\left(k+1\right)|\leq r|\Delta u_{i}\left(k\right)\right|$ satisfy for all k and r are a positive constant, where $\Delta u_{i}\left(k\right)=u_{i}\left(k\right)-u_{i}\left(k-1\right)$ and $\Delta y_{i}\left(k+1\right)=y_{i}\left(k+1\right)-y_{i}\left(k\right)$. Meanwhile the model (1) is generalized Lipschitz.*

**Remark** **1.** *The authors of [12,37] and those in their references have introduced the reasonability of Assumptions 1 and 2 for practical nonlinear systems and MASs.*

**Lemma** **1.** *Under these circumstances where the agent’s dynamic (1) satisfies Assumptions 1, 2, and $\Delta u_{i}\left(k\right)\neq 0$, the system (1) can utilize the following compact form linearization model to present [37,38].*

(2) $$\Delta y_{i}\left(k+1\right)=\Gamma_{i}\left(k\right)\Delta u_{i}\left(k\right)$$

*where $\left|\Gamma_{i}\left(k\right)\right|\leq \bar{r}$, $\bar{r}$ is a positive constant, and $\Gamma_{i}\left(k\right)$ is a variable named pseudo-partial-derivative (PPD).*

**Remark** **2.** *Using PPD to establish a dynamic linearization data model is called the PPD approach, where the PPD is only dependent on $\Delta y_{i}\left(k+1\right)$ and $\Delta u_{i}\left(k\right)$. Moreover, the dynamic linearization data model is updated by the PPD, which could approximate the practical dynamics of the controlled plant better. $\Gamma_{i}\left(k\right)$ is not easy to obtain, so we design a parameter estimation law (4) to obtained the estimation (${\hat{\Gamma }}_{i}\left(k\right)$) of $\Gamma_{i}\left(k\right)$. Meanwhile, the estimation error of $\Gamma_{i}\left(k\right)$ is analyzed in Theorem 1. Since the PPD approach is not complex and the dynamic linearization data model obtained is simple, the PPD approach is a hot topic in data-driven control for researches to study discrete-time nonlinear systems. However, it is still an open topic for utilizing the PPD approach to solve consensus problems of multi-agent systems, especially the multi-agent systems bipartite consensus problems with switching topologies.*

**Definition** **2.** *The following distributed measurement output:*

(3) $$\xi_{i}(k)=\sum_{j\in N_{i}}\left|a_{ij}\left(k\right)|(sign(a_{ij}\left(k\right))y_{j}(k)-y_{i}(k)\right)\;+b_{i}\left(k\right)(s_{i}\left(k\right)y_{0}(k)-y_{i}(k))$$

*If the agent i can directly get the desired trajectory from virtual leader 0, $i.e.,\left\{0,i\right\}\in \bar{E}$, $b_{i}\left(k\right)=1$. Otherwise, $b_{i}\left(k\right)=0$. Let $\varepsilon_{i}\left(k\right)=s_{i}y_{0}\left(k\right)-y_{i}\left(k\right)$ denote the tracking error, where $s_{i}=1$ for $i\in V_{1}$ and $s_{i}=-1$ for $i\in V_{2}$.*

**Assumption** **3.** *All of the time-varying switching communication graphs are strongly connected graphs and the trajectory information of the virtual leader can be transmitted to one or more follower agents directly.*

**Assumption** **4.** *In the relative literature, $\Gamma_{i}\left(k\right)>0\;,\;i=1,2,3,\ldots ,N$ ($or\;\Gamma_{i}\left(k\right)<0$) stratify for all k, so we assume $\Gamma_{i}\left(k\right)>0$ in this paper.*

**Remark** **3.** *The above Assumption 3 is a fundamental condition for researching the bipartite consensus tracking problems. Moreover, it can obviously find Assumption 4, which is implied in the traditional model-based control algorithms as a type of linear-like characteristic. Furthermore, this assumption is wildly used in some practical multi-agent systems, for instance, in unmanned air vehicles and mobile robots.*

## 3. Main Results

In order to solve the bipartite consensus tracking problem stated in Section 2.2, we propose the DMFABCT approach below:

(4) $${\hat{\Gamma }}_{i}(k)={\hat{\Gamma }}_{i}(k-1)+\frac{p\Delta u_{i}(k-1)}{w+|\Delta u_{i}(k-1){|}^{2}}(\Delta y_{i}(k)-{\hat{\Gamma }}_{i}\left(k-1\right)\Delta u_{i}(k-1))\;$$

(5) $${\hat{\Gamma }}_{i}(k)={\hat{\Gamma }}_{i}(1),\;\;\;\;\{\begin{array}{c} |{\hat{\Gamma }}_{i}(k)|\leq c\\ sign({\hat{\Gamma }}_{i}(k))\neq \;sign({\hat{\Gamma }}_{i}(1)) \end{array}$$

(6) $$u_{i}(k)=u_{i}(k-1)+\frac{\rho {\hat{\Gamma }}_{i}(k)}{\lambda +|{\hat{\Gamma }}_{i}(k){|}^{2}}\zeta_{i}(k)$$

where p>0, ρ>0 are the step sizes, which will be defined in the next section.w>0 and λ>0 are weight factors. According to Assumption 4, let ${\hat{\Gamma }}_{i}(1)>0$, which is the initial value of ${\hat{\Gamma }}_{i}(k)$, and it is the estimated value of $\Gamma_{i}(k)$. Practically, if the c is very small, it means that the ${\hat{\Gamma }}_{i}(k)$ does not update any more, thus, c is selected as 10^−4^.

**Remark** **4.** *It is noted that ${\hat{\Gamma }}_{i}(k)$ could be obtained by merely using the output data $\Delta y_{i}\left(k\right)$ in the parameters estimation scheme (4) and another important thing is worth pointing out that the convergence of parameters estimation scheme (4) can be guaranteed as shown in [12] and [37]. The control law (6) illustrates that the controlling input $u_{i}(k)$ is updated by using the distributed measurement output $\xi_{i}(k)$ for agent i, so that the algorithm is a kind of DMFABCT scheme.*

**Remark** **5.** *The feature of this DMFABCT scheme is that agents’ model dynamics are not required, for instance, the PPD parameters estimation algorithm is only used on the measured I/O data of multi-agent systems to complete the formulation, therefore, it is a classic data-driven control approach for solving the MASs’ BC problem.*

**Remark** **6.** *Both λ and ρ are important parameters of the distributed DMFABCT algorithms. A suitable λ, which is a weight parameter, can ensure the stability of MASs, and ρ is a controller parameter that can guarantee the tracking error that will be cut. Furthermore, the value ranges of ρ will be analyzed in the following Theorems.*

To analyze the stability of MASs, Lemma 2 is one of the important conditions.

**Lemma** **2.** *A time-varying irreducible substochastic matrix and the set of all possible T(Q) are denoted by T(Q) and T respectively [39]. Also, the diagonal entries of T(Q) are positive. Then, we can obtain*

‖T(Q)T(Q-1)⋅⋅⋅T(1)‖≤ϒ

*where 0<ϒ<1 and T(Q), K=1,2,…,Q, are Q matrices arbitrarily selected from T.*

The stability analysis of the DMFABCT approach is presented by Theorem 1.

**Theorem** **1.** *Under these circumstances where the MASs (1) satisfies Assumptions 1, 2, and 4 and its communication topology satisfies Assumption 3, apply the proposed DMFABCT algorithms (4)–(6) to track the desired reference trajectory $y_{0}(k)$, which is time invariable, i.e., $y_{0}(k)=const$, if ρ satisfies the following condition*

$$\rho <\frac{1}{\max=1,\ldots ,N,\;l=1,\ldots ,\kappa \;\sum_{j=1}^{N}\left|a_{ij}^{l}\left(k\right)\right|+b_{i}^{l}\left(k\right)}$$

*and $\lambda >\lambda_{\min}>0$, $\underset{k->\infty }{lim}\left\|\varepsilon_{i}(k)\right\|=0,\;i=1,2,\ldots ,N$.*

**Proof:**  We prove this theorem using the three steps below.

**Step 1** (Proving the Boundedness of ${\hat{\Gamma }}_{i}(k)$): Define ${\tilde{\Gamma }}_{i}(k)={\hat{\Gamma }}_{i}(k)-\Gamma_{i}(k)$. According to the Lemma 1 and parameter estimation law (4), the following equation can be obtained.

(7) $$\begin{array}{ll} {\tilde{\Gamma }}_{i}(k) & ={\hat{\Gamma }}_{i}(k)-\Gamma_{i}(k)\\ & =\;{\hat{\Gamma }}_{i}(k-1)+\frac{p\Delta u_{i}(k-1)}{w+|\Delta u_{i}(k-1){|}^{2}}(\Delta y_{i}(k)-{\hat{\Gamma }}_{i}\left(k-1\right)\Delta u_{i}(k-1))-\Gamma_{i}(k)\\ & =\frac{p\Delta u_{i}{\left(k-1\right)}^{2}}{w+|\Delta u_{i}(k-1){|}^{2}}(\Gamma_{i}(k-1)-{\hat{\Gamma }}_{i}(k-1))+\Gamma_{i}(k-1)-\Gamma_{i}(k)\\ & =(1-\frac{p\Delta u_{i}{\left(k-1\right)}^{2}}{w+|\Delta u_{i}(k-1){|}^{2}}){\tilde{\Gamma }}_{i}(k-1)+\Gamma_{i}(k-1)-\Gamma_{i}(k) \end{array}$$

According to Equation (7) the following equation can be obtained.

(8) $$\left|{\tilde{\Gamma }}_{i}(k)\right|\leq \left|\left(1-\frac{p\Delta u_{i}{\left(k-1\right)}^{2}}{w+|\Delta u_{i}(k-1){|}^{2}}\right)\right|\left|{\tilde{\Gamma }}_{i}(k-1)\right|\;+\left|\Gamma_{i}(k-1)-\Gamma_{i}(k)\right|.$$

The inequalities $p\Delta u_{i}{\left(k-1\right)}^{2}\leq {\left|\Delta u_{i}(k-1)\right|}^{2}\leq w+{\left|\Delta u_{i}(k-1)\right|}^{2}$ can be obtained by selecting p and w, which satisfy 0<p≤1 and w≥0. $\Delta u_{i}{\left(k-1\right)}^{2}={\left|\Delta u_{i}(k-1)\right|}^{2}$ because the system studied in this paper is a single input and output. Thus, a constant ϖ can be selected to satisfy the following inequality.

(9) $$0<\left|\left(1-\frac{p\Delta u_{i}{\left(k-1\right)}^{2}}{w+{\left|\Delta u_{i}(k-1)\right|}^{2}}\right)\right|\leq \varpi <1$$

Since $\left|\Gamma_{i}(k)\right|\leq \bar{r}$, according to Assumption 4, the following inequalities can be obtained.

$$\{\begin{array}{c} \Gamma_{i}\left(k\right)-\Gamma_{i}\left(k-1\right)\leq \Gamma_{i}\left(k\right)\leq \bar{r},\;\mathrm{if}\;\Gamma_{i}\left(k\right)\leq \Gamma_{i}\left(k-1\right)\\ \Gamma_{i}\left(k-1\right)-\Gamma_{i}\left(k\right)\leq \Gamma_{i}\left(k\right)\leq \bar{r},\;\mathrm{if}\;\Gamma_{i}\left(k-1\right)\leq \Gamma_{i}\left(k\right) \end{array}$$

Obviously, it can obtain $\left|\Gamma_{i}(k-1)-\Gamma_{i}(k)\right|\leq \bar{r}$ and

(10) $$\begin{array}{ll} \left|{\tilde{\Gamma }}_{i}(k)\right|\leq & \varpi \left|{\tilde{\Gamma }}_{i}(k-1)\right|+\bar{r}\\ & \leq \varpi^{2}\left|{\tilde{\Gamma }}_{i}(k-2)\right|+\varpi \bar{r}+\bar{r}\\ & \leq \varpi^{3}\left|{\tilde{\Gamma }}_{i}(k-3)\right|+\varpi^{2}\bar{r}+\varpi r+\bar{r}\\ & \leq \cdot \cdot \cdot \leq \varpi^{k-1}\left|{\tilde{\Gamma }}_{i}(1)\right|+\varpi^{k-2}\bar{r}+\cdot \cdot \cdot +\varpi \bar{r}+\bar{r}\\ & \leq \varpi^{k-1}\left|{\tilde{\Gamma }}_{i}(1)\right|+\frac{\bar{r}\left(1-\varpi^{k-1}\right)}{1-\varpi } \end{array}$$

so that $\underset{k->\infty }{\lim}\left|{\tilde{\Gamma }}_{i}(k)\right|=\frac{\bar{r}}{1-\varpi }$. Moreover, since $\Gamma_{i}(k)$ is bounded, it is obvious that ${\hat{\Gamma }}_{i}(k)$ is bounded.

**Step 2** (Proving the Convergence of ε(k)): Since $\varepsilon_{i}\left(k\right)=s_{i}\left(k\right)y_{0}\left(k\right)-y_{i}\left(k\right)$, Equation (3) can be rewritten as follows:

(11) $$\xi_{i}(k)=\sum_{j\in N_{i}}|a_{ij}\left(k\right)|\left(\left(sign(a_{ij}\left(k\right)\right)y_{j}\left(k\right)-y_{i}\left(k\right)\right)\;+b_{i}\left(k\right)\varepsilon_{i}\left(k\right)$$

Equation (11) can be written for clarity as a compact form

(12) $$\begin{array}{ll} \xi (k) & ={\left[\xi_{1}(k),\xi_{2}(k),\cdot \cdot \cdot ,\xi_{N}(k)\right]}^{T}\\ & =-L\left(k\right)y(k)+B\left(k\right)(s\left(k\right){\bar{y}}_{0}(k)-y(k))\\ & =-L\left(k\right)y(k)+Ls\left(k\right){\bar{y}}_{0}(k)+B\left(k\right)(s\left(k\right){\bar{y}}_{0}(k)-y(k))\\ & =\left(B\left(k\right)+L\left(k\right)\right)\left(s\left(k\right){\bar{y}}_{0}\left(k\right)-y\left(k\right)\right)\\ & =\left(B\left(k\right)+L\left(k\right)\right)\varepsilon \left(k\right) \end{array}$$

where

$$B\left(k\right)=diag\left(b_{1}\left(k\right),b_{2}\left(k\right),\cdot \cdot \cdot ,b_{N}\left(k\right)\right)$$

$$\varepsilon \left(k\right)={\left[\varepsilon_{1}\left(k\right),\varepsilon_{2}\left(k\right),\cdot \cdot \cdot ,\varepsilon_{N}\left(k\right)\right]}^{T}$$

$$s\left(k\right)=diag\left(s_{1}\left(k\right),s_{2}\left(k\right),\cdot \cdot \cdot ,s_{N}\left(k\right)\right)$$

$s_{i}=1$ for $i\in V_{1}$ and $s_{i}=-1$ for $i\in V_{2}$, ${\bar{y}}_{0}=1⊗y_{0}$, and $1=col\left(1,\cdot \cdot \cdot ,1\right)\in R^{N}$ is the N-vector. Moreover, obviously $Ls\left(p\right){\bar{y}}_{0}(k)=0$.

According to Equation (12), the compact form of the DMFABCT algorithm (6) can be written as follows:

(13) $$\begin{array}{ll} u\left(k\right) & ={\left[u_{1}\left(k\right),u_{2}\left(k\right),\cdot \cdot \cdot ,u_{N}\left(k\right)\right]}^{T}\\ & =u(k-1)+\rho \Omega_{1}(k)\xi (k)\\ & =u\left(k-1\right)+\rho \Omega_{1}\left(k\right)\left(L\left(k\right)+B\left(k\right)\right)\varepsilon \left(k\right) \end{array}$$

where

$$\Omega_{1}\left(k\right)=\mathrm{diag}\left(\frac{{\hat{\Gamma }}_{1}\left(k\right)}{\lambda +|{\hat{\Gamma }}_{1}\left(k\right){|}^{2}},\cdot \cdot \cdot ,\frac{{\hat{\Gamma }}_{N}\left(k\right)}{\lambda +|{\hat{\Gamma }}_{N}\left(k\right){|}^{2}}\right)$$

According to equations $\Delta y_{i}\left(k+1\right)=\Gamma_{i}\left(k\right)\Delta u_{i}\left(k\right)$, $\Delta y_{i}\left(k+1\right)=y_{i}\left(k+1\right)-y_{i}\left(k\right)$, and $\Delta u_{i}\left(k\right)=u_{i}\left(k\right)-u_{i}\left(k-1\right)$, Equation (2) can be written as follows:

(14) $$\begin{array}{ll} y\left(k+1\right) & =y\left(k\right)+\Omega_{T}\left(k\right)\Delta u\left(k\right)\\ & =y\left(k\right)+\Omega_{T}\left(k\right)\left(u\left(k\right)-u\left(k-1\right)\right)\\ & =y\left(k\right)+\Omega_{T}\left(k\right)(u\left(k-1\right)+\rho \Omega_{1}\left(k\right)\left(L\left(k\right)+B\left(k\right)\right)\varepsilon \left(k\right)-u\left(k-1\right))\\ & =y\left(k\right)+\rho \Omega_{1}\left(k\right)\Omega_{T}\left(k\right)\left(L\left(k\right)+B\left(k\right)\right)\varepsilon \left(k\right) \end{array}$$

where $\Omega_{T}\left(k\right)=\mathrm{diag}\left({\hat{\Gamma }}_{1}\left(k\right),{\hat{\Gamma }}_{2}\left(k\right),\cdot \cdot \cdot ,{\hat{\Gamma }}_{N}\left(k\right)\right)$. According to $\varepsilon \left(k\right)=s\left(p\right){\bar{y}}_{0}\left(k\right)-y\left(k\right)$, it is easy to get ε(k+1)−ε(k)=y(k)−y(k+1). Furthermore, we could substitute (13) to (14) to get

(15) $$\begin{array}{ll} \varepsilon \left(k+1\right) & =\varepsilon \left(k\right)-\rho \Omega_{1}\left(k\right)\Omega_{T}\left(k\right)\left(L\left(k\right)+B\left(k\right)\right)\varepsilon \left(k\right)\\ & =\left(I-\rho \Psi \left(k\right)\left(L\left(k\right)+B\left(k\right)\right)\right)\varepsilon \left(k\right)\\ & =\left(I-\rho \Omega_{1}\left(k\right)\Omega_{T}\left(k\right)\left(L\left(k\right)+B\left(k\right)\right)\right)\varepsilon \left(k\right)\\ & =\left(I-\rho \Xi \left(k\right)\right)\varepsilon \left(k\right) \end{array}$$

where $\Psi \left(k\right)=\Omega_{1}\left(k\right)\Omega_{T}\left(k\right)=diag\left(\Phi_{1}\left(k\right),\Phi_{2}\left(k\right),\cdot \cdot \cdot ,\Phi_{n}\left(k\right)\right)$, $\Phi_{i}\left(k\right)=\frac{\Gamma_{i}\left(k\right){\hat{\Gamma }}_{i}\left(k\right)}{\lambda +|{\hat{\Gamma }}_{i}\left(k\right){|}^{2}},\;i=1,2,\ldots ,N$, Ξ(k)=Ψ(k)(L(k)+B(k)). From (15), we can obtain that if ‖I−ρΞ(k)‖<1 for all k, then ${lim}_{k->\infty }\|\varepsilon \left(k+1\right)\|=0$.

Step 3 (Obtaining the Convergence Condition of MASs): In this step, the convergence condition of MASs will be derived.

According to the conditions $\Gamma_{i}(k)\leq \bar{r}$, $sign({\hat{\Gamma }}_{i}(k))=sign({\hat{\Gamma }}_{i}(1))>0$, $\;\lambda +|{\hat{\Gamma }}_{i}(k){|}^{2}\geq 2\sqrt{\lambda }|{\hat{\Gamma }}_{i}(k)|$, $\lambda_{min}>0$, and $\lambda >\lambda_{min}$ for all i=1,2,…,N, the following inequalities can be obtained:

$$0<\frac{\Gamma_{i}(k){\hat{\Gamma }}_{i}(k)}{\lambda +|{\hat{\Gamma }}_{i}(k){|}^{2}}\leq \frac{\bar{r}{\hat{\Gamma }}_{i}(k)}{2\sqrt{\lambda }|{\hat{\Gamma }}_{i}(k)|}<\frac{\bar{r}}{2\sqrt{\lambda }}<\frac{\bar{r}}{2\sqrt{\lambda_{\min}}}<1$$

First of all, in order to guarantee the strictly connected property of MASs under all of the communication topologies, I−ρΞ(k) must be an irreducible matrix. Secondly, $0<\Phi_{i}\left(k\right)<1$ for all i=1,2,…,N and ρ satisfies following inequality

$$\rho <\frac{1}{\max=1,\ldots ,N,\;l=1,\ldots ,\kappa \;\sum_{j=1}^{N}\left|a_{ij}^{l}\left(k\right)\right|+b_{i}^{l}\left(k\right)},$$

which means that all of the diagonal entry in L(k)+B(k) are larger than the reciprocal of ρ. In this case, obviously I−ρΞ(k) is strictly less than one, so I−ρΞ(k) is an irreducible substochastic matrix and its diagonal entries are positive. According to (15), the following inequality can be obtained.

(16) $$\begin{array}{ll} \varepsilon (k+1) & =(I-\rho \Xi (k))\varepsilon (k)\\ & \leq \left\|I-\rho \Xi (k)\right\|\left\|\varepsilon (k)\right\|\\ & \leq \left\|I-\rho \Xi (k)\right\|\left\|I-\rho \Xi (k-1)\right\|\left\|\varepsilon (k-1)\right\|\\ & \leq \left\|I-\rho \Xi (k)\right\|\left\|I-\rho \Xi (k-1)\right\|\left\|\varepsilon (k-1)\right\|\cdot \cdot \cdot \left\|I-\rho \Xi (1)\right\|\left\|\varepsilon (1)\right\| \end{array}$$

According to Lemma 1, the following inequality can be obtained.

$$\left\|\varepsilon (k+1)\right\|\leq {ϒ}^{\left\lfloor \frac{k}{Q}\right\rfloor }\left\|\varepsilon (1)\right\|\;\;\;\;$$

where ⌊·⌋ stands for the floor function. Hence, the bipartite consensus fixed trajectory tracking errors of MASs can converge to the origin. □

**Theorem** **2** *Under these circumstances where the MASs (1) satisfies Assumptions 1, 2, and 4 and its communication topology satisfies Assumption 3, apply the designed DMFBAC schemes (4)–(6) to track the time-varying reference trajectory $y_{0}\left(k\right)$, where ${\bar{y}}_{0}\left(k\right)={\left[y_{0}\left(k\right),y_{0}\left(k\right),\cdot \cdot \cdot ,y_{0}\left(k\right)\right]}^{T}$ and $\Delta {\bar{y}}_{0}(k)={\bar{y}}_{0}(k+1)-{\bar{y}}_{0}(k)$. Moreover, if ρ satisfies the following condition*

$$\rho <\frac{1}{\max=1,\ldots ,N,\;l=1,\ldots ,\kappa \;\sum_{j=1}^{N}\left|a_{ij}^{l}\left(k\right)\right|+b_{i}^{l}\left(k\right)}$$

$\|\Delta {\bar{y}}_{0}(k)\|<r_{y}$ *and $\lambda >\lambda_{min}>0$, then there will be a small constant α, where $\underset{k->\infty }{lim}\left\|\varepsilon_{i}(k)\right\|\leq \alpha ,\;i=1,2,\ldots ,N$. The value of α is dependent on output gain of the time-varying trajectory.*

**Proof:**  Since $\varepsilon (k)=s\left(k\right){\bar{y}}_{0}(k)-y(k)\;\;\;$, then ε(k+1)−ε(k)=y(k)  −y(k−1) , so that the bipartite consensus tracking error Equation in (15) can be rewritten as

(17) $$\varepsilon \left(k+1\right)=\left(I-\rho \Xi \left(k\right)\right)\varepsilon \left(k\right)+\Delta {\bar{y}}_{0}\left(k\right)$$

so that the following inequality can be obtained.

(18) $$\begin{array}{ll} \left\|\varepsilon (k+1)\right\| & \leq \left\|I-\rho \Xi (k)\right\|\left\|\varepsilon (k)\right\|+\left\|\Delta {\bar{y}}_{0}(k)\right\|\\ & \leq \left\|I-\rho \Xi (k)\right\|\left\|I-\rho \Xi (k-1)\right\|\left\|\varepsilon (k-1)\right\|\;+\left\|I-\rho \Xi (k)\right\|\left\|\Delta {\bar{y}}_{0}(k-1)\right\|+\left\|\Delta {\bar{y}}_{0}(k)\right\|\\ & =\left\|I-\rho \Xi (k)\right\|\left\|I-\rho \Xi (k-1)\right\|\cdot \cdot \cdot \left\|I-\rho \Xi (1)\right\|\left\|\varepsilon (1)\right\|+\left\|\Delta {\bar{y}}_{0}(k)\right\|+\left\|I-\rho \Xi (k)\right\|\left\|\Delta {\bar{y}}_{0}(k-1)\right\|+\cdot \cdot \cdot \\ & +\left\|I-\rho \Xi (k)\right\|\left\|I-\rho \Xi (2)\right\|\left\|\Delta {\bar{y}}_{0}(1)\right\|\\ & \leq \left\|I-\rho \Xi (k)\right\|\left\|I-\rho \Xi (k-1)\right\|\cdot \cdot \cdot \left\|I-\rho \Xi (1)\right\|\left\|\varepsilon (1)\right\|\;+r_{y}+\left\|I-\rho \Xi (k)\right\|r_{y}\\ & +\left\|I-\rho \Xi (k)\right\|\left\|I-\rho \Xi (k-1)\right\|r_{y}+\cdot \cdot \cdot +\left\|I-\rho \Xi (k)\right\|\cdot \cdot \cdot \left\|I-\rho \Xi (2)\right\|r_{y}\;\; \end{array}$$

Let $O\left(K\right)={ϒ}^{\left\lfloor KQ/Q\right\rfloor }+{ϒ}^{\left\lfloor KQ+1/Q\right\rfloor }+\cdot \cdot \cdot +{ϒ}^{\left\lfloor ((K+1)Q-1)/Q\right\rfloor }\;$ and utilizing Lemma 1 we can obtain that $O(k)=Q{ϒ}^{k}$, and (16) can be written as follows:

(19) $$\begin{array}{ll} \underset{k->\infty }{\lim}\left\|\varepsilon (k+1)\right\| & =\underset{k->\infty }{\lim}\left({ϒ}^{\left\lfloor \frac{K}{Q}\right\rfloor }\left\|\varepsilon (1)\right\|+\left({ϒ}^{\left\lfloor \frac{K-1}{Q}\right\rfloor }+{ϒ}^{\left\lfloor \frac{K-2}{Q}\right\rfloor }+\cdot \cdot \cdot +{ϒ}^{\left\lfloor \frac{0}{Q}\right\rfloor }\right)r_{y}\right)\\ & =\underset{k->\infty }{\lim}\left({ϒ}^{\left\lfloor \frac{K-1}{Q}\right\rfloor }+{ϒ}^{\left\lfloor \frac{K-2}{Q}\right\rfloor }+\cdot \cdot \cdot +{ϒ}^{\left\lfloor \frac{0}{Q}\right\rfloor }\right)r_{y}\\ & =\underset{k->\infty }{\lim}\left({ϒ}^{\left\lfloor \frac{(K+1)Q-1}{Q}\right\rfloor }+{ϒ}^{\left\lfloor \frac{(K+1)Q-2}{Q}\right\rfloor }+\cdot \cdot \cdot +{ϒ}^{\left\lfloor \frac{K-1}{Q}\right\rfloor }+\cdot \cdot \cdot +{ϒ}^{\left\lfloor \frac{0}{Q}\right\rfloor }\right)r_{y}\\ & =\underset{k->\infty }{\lim}\left(O(k)+O(k-1)+\cdot \cdot \cdot +O(0)\right)r_{y}\\ & =Q\underset{k->\infty }{\lim}\left({ϒ}^{k}+{ϒ}^{k-1}+\cdot \cdot \cdot +{ϒ}^{0}\right)r_{y}\\ & =\frac{Q}{1-ϒ}r_{y} \end{array}$$

where ⌊·⌋ denotes the floor function. Finally, the bounded of ‖ε(k+1)‖ is obtained.

Thus, bipartite time-varying trajectory tracking error is bound, which is dependent on the output gain $\left\|\Delta {\bar{y}}_{0}(k)\right\|$ of the reference trajectory. □

## 4. Simulation

In order to illustrate the efficiency of the proposed bipartite consensus tracking algorithm, three numerical simulations with seven follower agents are performed, where agents are governed by

$$\begin{array}{c} Agent1:y_{1}(k+1)=\frac{y_{1}(k)u_{1}(k)}{1+y_{1}^{3}(k)}+0.5u_{1}(k),\\ Agent2:y_{2}(k+1)=\frac{y_{2}(k)u_{2}(k)}{1+y_{2}^{3}(k)}+0.45u_{2}(k),\\ Agent3:y_{3}(k+1)=\frac{y_{3}(k)u_{3}(k)}{1+y_{3}^{5}(k)}+0.7u_{3}(k),\\ Agent4:y_{4}(k+1)=\frac{y_{4}(k)u_{4}(k)}{1+y_{4}^{5}(k)}+0.6u_{4}(k), \end{array}$$

$$\begin{array}{c} Agent5:y_{5}(k+1)=\frac{y_{5}(k)u_{5}(k)}{1{+y}_{5}^{7}(k)}+0.9u_{5}(k),\\ Agent6:y_{6}(k+1)=\frac{y_{5}(k)u_{5}(k)}{1{+y}_{5}^{7}(k)}+0.75u_{5}(k),\\ Agent7:y_{7}(k+1)=\frac{y_{5}(k)u_{5}(k)}{1{+y}_{5}^{7}(k)}+0.65u_{5}(k). \end{array}$$

It can be discovered that each agent has a unique dynamics system model, so the considered MASs are heterogeneous. Furthermore, it is noteworthy that the above dynamics system models are only applied to produce the I/O data for the MASs, while the distributed DMFABCT algorithm does not utilize any model information. During the design of this algorithm, the dynamics of MASs are all unknown.

**Figure 1:**
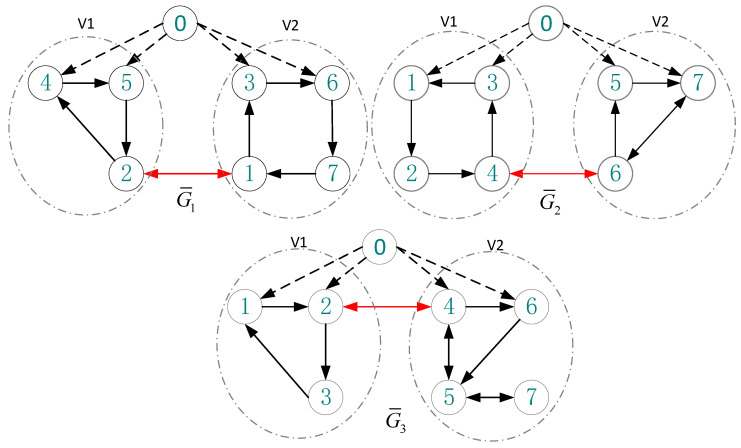
Communication topology among agents.

The communication topology of considered MASs is shown in Figure 1. It demonstrates that the virtual leader is denoted by using vertex 0 and the followers are distributed into two alliances in each topology. Moreover, in Figure 1, the black solid lines are used to express the cooperative relationships among agents, and the competitive relationships are denoted by dotted lines. It is noted that only a subset of agents could directly receive the information from the leader. Moreover, the information among agents only transmits along the arrows and the direction is fixed. Although other agents cannot directly get the commands from the virtual leader, all of the communication graphs satisfy Assumption 3, so the virtual leader can intervene in the two competitive alliances. As the matrixes above show, the reciprocal of the greatest diagonal entry of L(l)+B(l) is 0.5 for l=1,2,3. In order to satisfy the convergence condition for all i=1,2,3,4,5,6,7 in Theorem 2, we choose the controller parameters as  ρ=0.3 for each simulation and the other parameters are selected as p=0.5, w=1, λ=0.5, and $c=10^{-4}$.

### 4.1. Fixed Trajectory Tracking Example

In order to obtain a clear result of this simulation, a piecewise function and the desired reference trajectory are given below:

$$\{\begin{array}{cc} {\bar{G}}_{1}, & 0\leq k\leq 400\\ {\bar{G}}_{2}, & 400<k\leq 800\\ {\bar{G}}_{3}, & 800<k\leq 1400 \end{array}$$

$$y_{0}\left(k\right)\{\begin{array}{c} 10,\\ 20,\\ 15, \end{array}\;\;\begin{array}{c} 0<k<400\\ 400\leq k<800\\ 800\leq k<1400 \end{array}$$

Initial conditions are chosen as $u_{i}\left(1\right)=0$, ${\hat{\Gamma }}_{i}\left(1\right)=2$ for all agents and $y_{1}\left(1\right)=0.5$, $y_{2}\left(1\right)=3.5$, $y_{3}\left(1\right)=6.5$, $y_{4}\left(1\right)=4.5$, $y_{5}\left(1\right)=1.5$, $y_{6}\left(1\right)=5.5$, $y_{7}\left(1\right)=5.5$ in this simulation.

**Figure 2:**
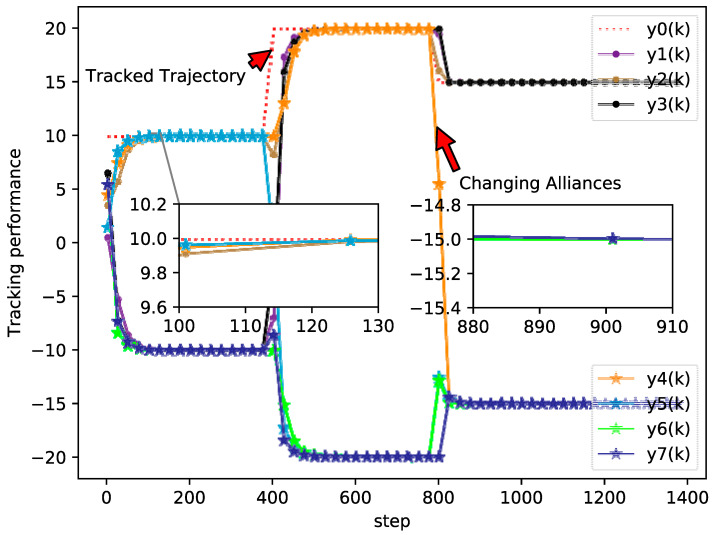
Tracking performance of each agent (example 1).

**Figure 3:**
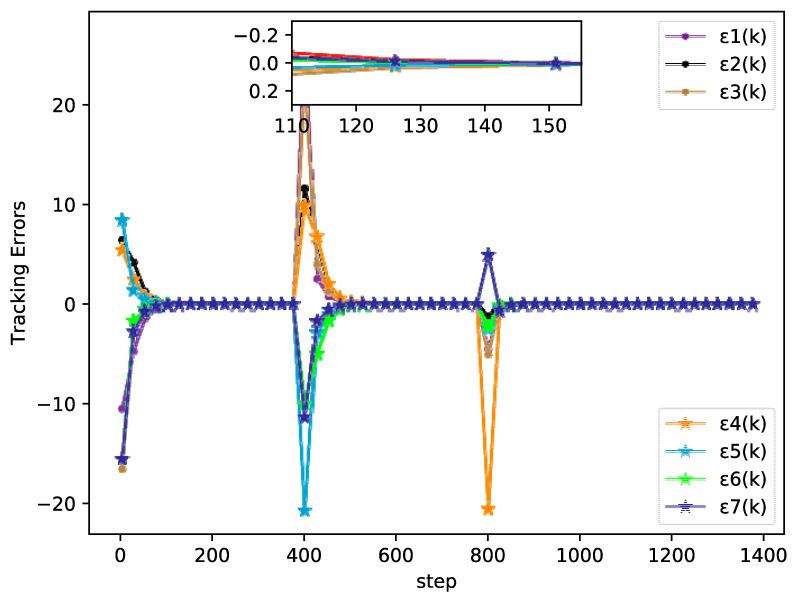
Tracking errors of each agent (example 1).

**Figure 4:**
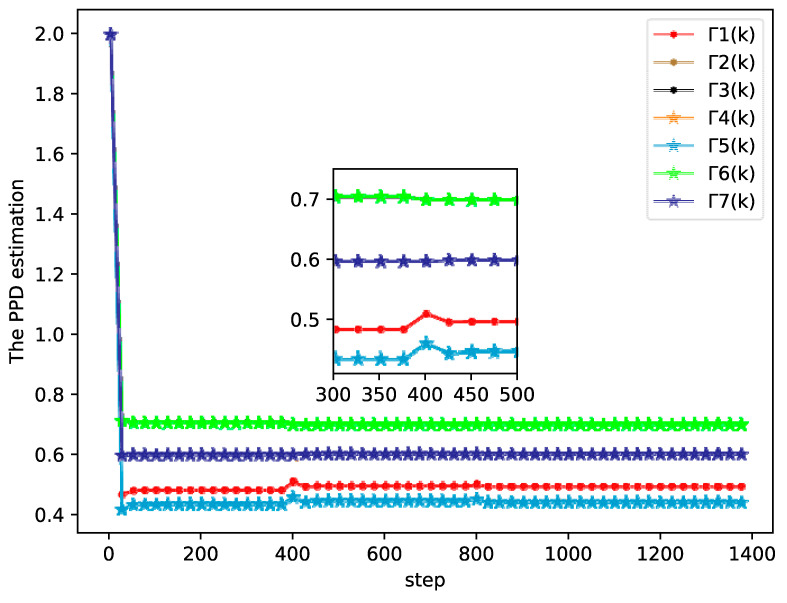
Pseudo partial derivative (PPD) estimation of each agent (example 1).

The simulation results of the bipartite tracking performance, tracking errors, and PPD estimation of each agent are shown in Figure 2, Figure 3 and Figure 4, respectively.

From Figure 2, Figure 3 and Figure 4 it can be seen that the output between followers and leader has an extreme variation initially, but the bipartite tracking errors can be decreased radically and the bipartite tracking is realized after a few steps. For example, in Figure 2, the value of trajectory is changed from 10 to 20 at k=400 and we could also find that several agents exchanged their groups at the same time, but only after about 100 steps after a new bipartite consensus is achieved, which Figure 3 also reveals. Furthermore, from Figure 4 we can see that the changing of the topology and the desire trajectory affect the estimation value of PPDs for each agent, but they achieve stable values immediately, which shows that the proposed DMFABCT has a good robustness.

### 4.2. Time-Varying Trajectory Tracking Example

In this example, the bipartite consensus time-varying trajectory tracking is discussed, and the desired trajectory is

$$y_{0}\left(k+1\right)=90\;cos\left(k\pi /\Psi \right)+100$$

where Ψ=2200 is the output gain rate and the time-varying topologies are governed by

$$\{\begin{array}{cc} {\bar{G}}_{1}, & 0\leq k\leq 2500\\ {\bar{G}}_{2}, & 2500<k\leq 5000\\ {\bar{G}}_{3}, & 5000<k\leq 8000 \end{array}$$

where the initial data of $y_{i}(k)$, $u_{i}(k)$, dynamics of each agent, and other parameters were defined in the beginning of this section.

**Figure 5:**
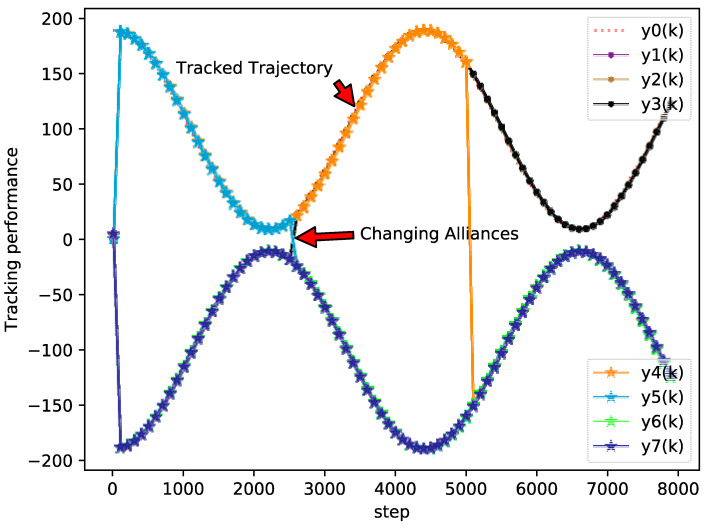
Tracking performance of each agent (example 2).

**Figure 6:**
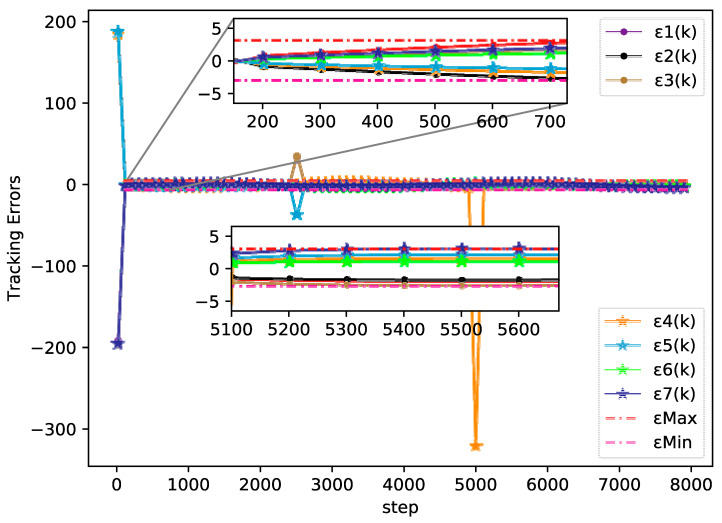
Tracking errors of each agent (example 2).

**Figure 7:**
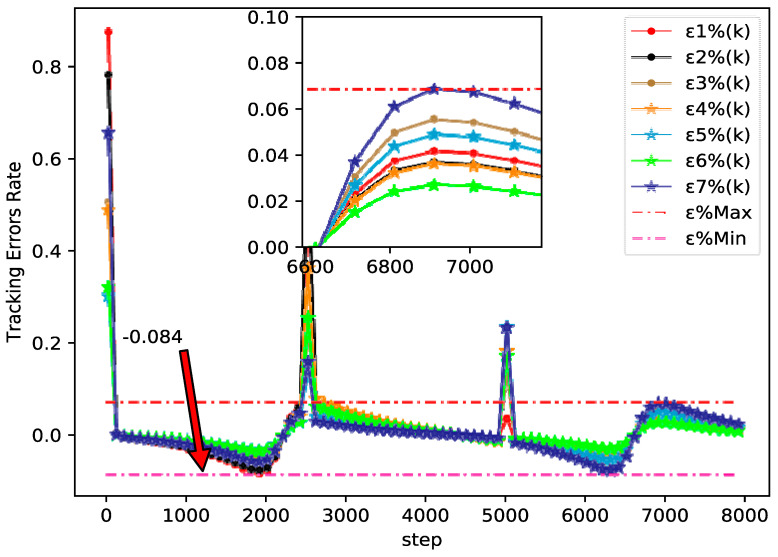
Tracking errors rate of each agent at Q=2200 (example 2).

**Figure 8:**
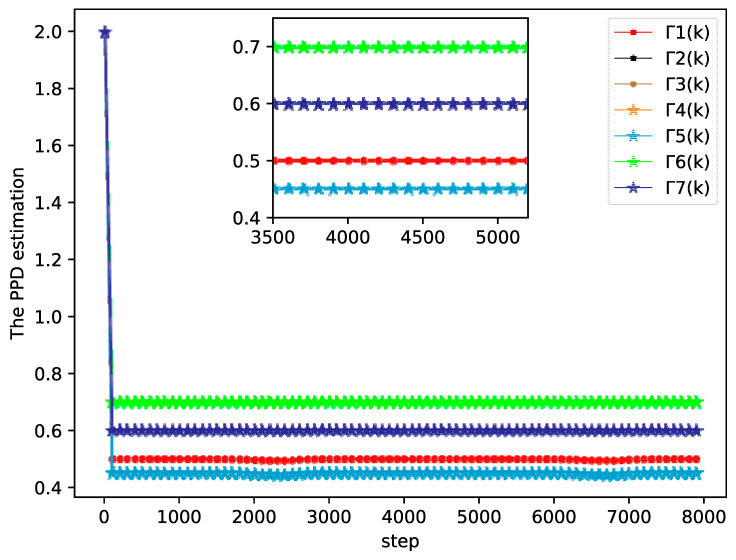
PPD estimation of each agent (example 2).

The bipartite consensus tracking performance of this example and the tracking errors of each agent are presented in Figure 5, which shows that the DMFABCT scheme can decrease the number of errors dramatically. Although the errors of the bipartite tracking cannot be removed, they converge to a small bound, which is demonstrated in Figure 6 and Figure 7. Compared with the desired output data of agents, the max distortion rate can be obtained in Figure 7, which is 0.084%. Obviously, this result demonstrates that MASs with switching topologies also can perform the bipartite time-varying tracking tasks. From Figure 8, we can also arrive at the same conclusion that MASs can change the value of PPDs to adaptive environmental change and can obtain a high fault-tolerance property.

**Figure 9:**
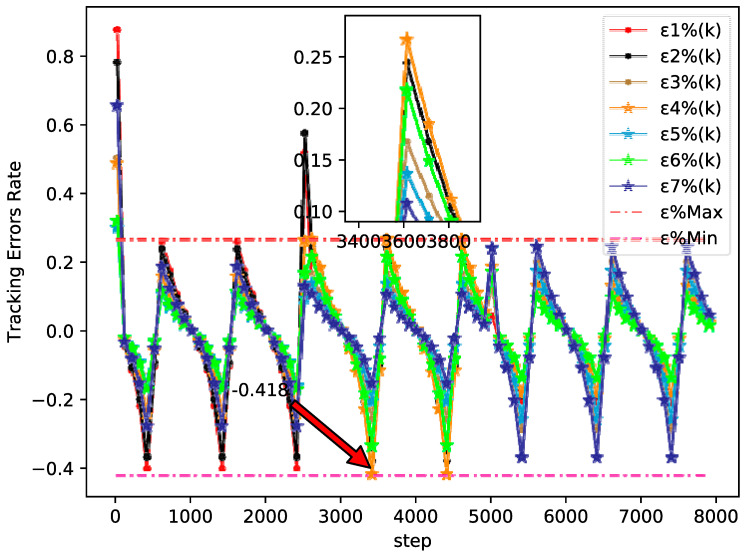
Tracking errors rate of each agent at Ψ=500 (example 2).

**Figure 10:**
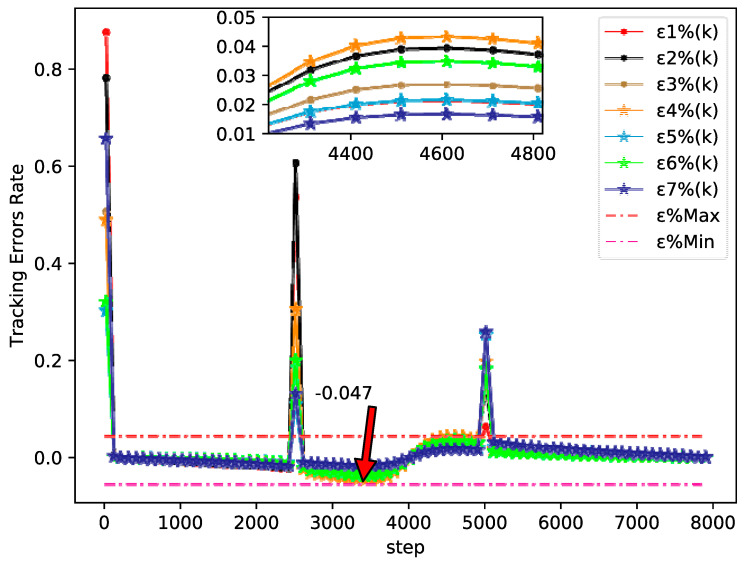
Tracking errors rate of each agent at Ψ=4000 (example 2).

**Figure 11:**
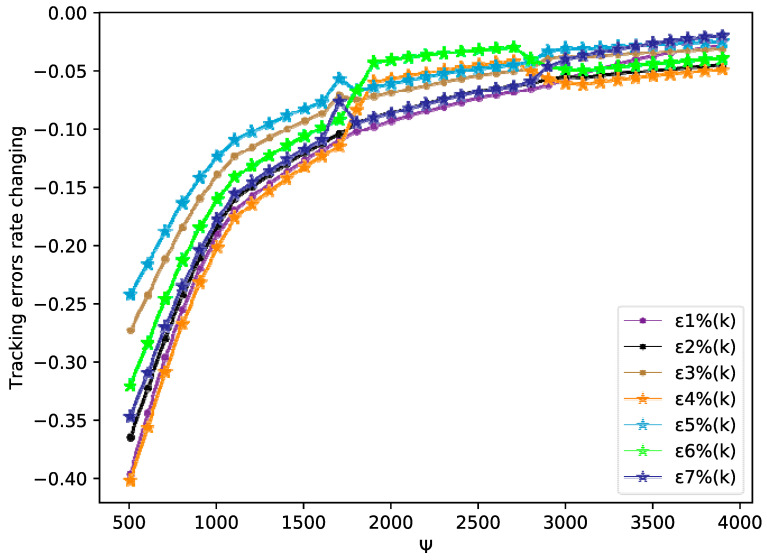
Tracking errors rate of each agent at Ψ∈[500,4000] (example 2).

By tracking performance of different tracking trajectories, according to Figure 3 and Figure 6, we can conclude that the performance of fixed trajectory tracking is better than that of the time-varying trajectory tracking, which further validates the correctness of the theoretical analysis in Section 3. In addition, in order to further analyze the errors forces of the time-varying trajectory, we change the output gain rate Ψ of the desired trajectory $y_{0}\left(k+1\right)=90\;cos\left(k\pi /\Psi \right)+100$ from 500 to 4000 to analyze the tracking performance. From Figure 9, we can easily find that the error rates of each agent all decrease, when the value of Ψ increases. The error rates of MASs at Ψ=500, Ψ=2200, and Ψ=4000 are shown in Figure 7, Figure 10 and Figure 11, respectively. Although the biggest error rate of MASs at Ψ=500 is about 0.418%, it can bind the error rates of each agent, which means that the errors of MASs are also bounded. Furthermore, errors rates of each agent, which are shown in Figure 11, are close to the original point, so that it further demonstrates the correctness of Theorem 2. Meanwhile, we can conclude that MASs are stable under the proposed DMFABCT scheme and the tracking errors are dependent on the output gain $\left\|\Delta {\bar{y}}_{0}(k)\right\|$ of the reference trajectory.

### 4.3. Realistic DC Linear Motors Example

In this case, we utilize seven permanent magnet DC linear motors to perform fixed and time-varying trajectory bipartite consensus tracking tasks. The realistic dynamic of the DC linear motor is investigated in [37,40], which has been modeled as below:

$$\left\{ \begin{array}{l} \dot{x}\left(t\right)=v\left(t\right)\\ v\left(t\right)=\frac{u\left(t\right)-f_{friction}\left(t\right)-f_{ripple}\left(t\right)}{m}\\ y\left(t\right)=v\left(t\right). \end{array}\right.$$

where t is continuous time (s), x(t) is the position (m), v(t) is the speed (m/s), m is the combined mass of translator and load, u(t) is the developed force (N), $f_{friction}\left(t\right)$ is the friction force (N), and $f_{ripple}\left(t\right)$ is the ripple force (N). The friction and ripple forces have been identified as:

ffriction(t)=(fc+(fs−fc)e−(x˙x˙δ)δ+fvx˙)sign(x˙)fripple(t)=b1sin(w0x(t))

where $f_{c}$ is the minimum level of Coulomb friction and $f_{s}$ is the level of static friction, ${\dot{x}}_{\delta }$ and $f_{v}$ are lubricant and load parameters, respectively. δ is an additional empirical parameter. In this example, these parameters are selected as: m=0.59kg, ${\dot{x}}_{\delta }=0.1$, δ=1, $f_{c}=10N$, $f_{s}=20N$, $f_{v}=10N\cdot s\cdot m^{-1}$, $b_{1}=8.5N$, $w_{0}=314s^{-1}$.The desired velocity is given as

$$y_{0}\left(t\right)=90\;cos\left(t\pi /4000\right)+100,\;t\in \left[0,8\right]$$

Using the Euler formula to discretize the above model and selecting sampling time as h=0.001, we have T=1000.

In this case, a random noise is introduced in the output measurement data for each DC motor. Moreover, we define the bound of the noise as [-0.02,0.02]. Here, we use the same parameters and the communication topology as those of example 2 to perform the simulation.

**Figure 12:**
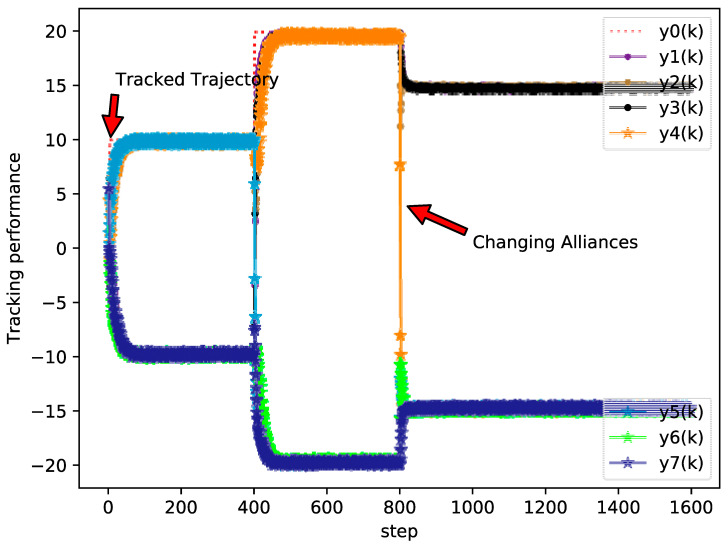
Tracking errors of each agent (example 3).

**Figure 13:**
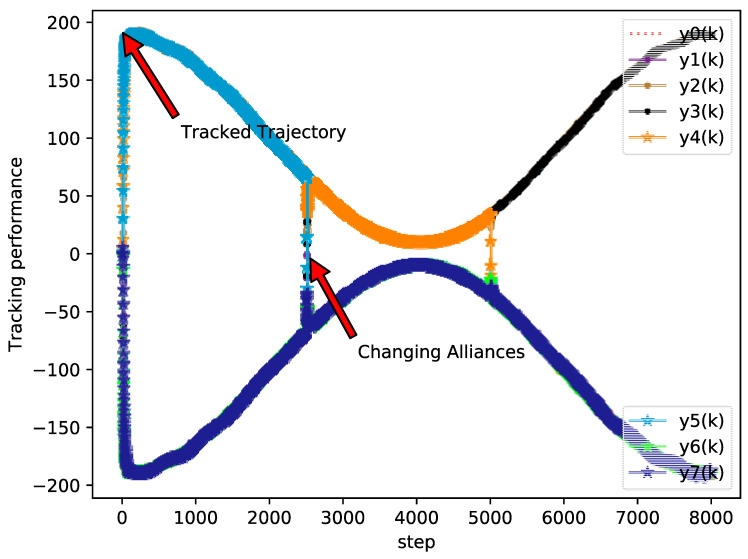
Tracking errors of each agent (example 4).

The fixed trajectory bipartite consensus tracking performances of seven DC motors are shown in Figure 12 and another tracking task is presented in Figure 13. From the two simulation results, we observe that several agents have changed their alliance, but the results of the two different bipartite consensus tracking tasks show that the tracking errors of MASs can be reduced, which further proves the effectiveness and applicability of the designed DMFABCT.

As shown above, the proposed DMFABCT scheme is correct and effective.

## 5. Conclusions

In this work, a data-driven bipartite consensus tracking scheme has been proposed for unknown nonlinear discrete-time multi-agent systems with switching topologies, and a compact form linearization model is established. This algorithm ensures that all agents can track the fixed and time-varying desired trajectory and realize the bipartite tracking. Compared with the model-based control algorithm, one of the main advanced features in our method is that it does not need the agent’s dynamics and requires only the input–output. Moreover, both of the cooperation and competition relationships among multi-agent systems are considered, and the convergence and stability of the algorithm are proven by rigorous mathematical analyses. Meanwhile, the corresponding simulation of the bipartite consensus tracking algorithm has been presented to validate the effectiveness of the proposed algorithm. In the future work, we will consider the bipartite consensus problem for multi-input-multi-output multi-agent systems with delay and disturbances.
